# Case report demonstrating novel approaches for leadless pacemaker implantation in the single ventricle heart

**DOI:** 10.1093/ehjcr/ytaf146

**Published:** 2025-03-28

**Authors:** Patrick Hayle, Fatima Altayeb, Angela Hale, Archana Rao, Reza Ashrafi

**Affiliations:** Department of Cardiology, Liverpool Heart and Chest Hospital, Thomas Drive, Liverpool L14 3PE, UK; Department of Cardiology, Liverpool Heart and Chest Hospital, Thomas Drive, Liverpool L14 3PE, UK; Department of Cardiology, Liverpool Heart and Chest Hospital, Thomas Drive, Liverpool L14 3PE, UK; Department of Cardiology, Liverpool Heart and Chest Hospital, Thomas Drive, Liverpool L14 3PE, UK; Department of Cardiology, Liverpool Heart and Chest Hospital, Thomas Drive, Liverpool L14 3PE, UK

**Keywords:** Leadless, Pacemaker, Fontan, Case Report

## Abstract

**Background:**

The congenital heart disease population can provide a unique set of challenges during pacemaker implant, including the necessity of transvenous leads in a young group of patients. In this case report we demonstrate how leadless pacemakers may be used as an option in patients with Fontan circulation.

**Case Summary:**

We present two cases of novel use of the Abbott AVEIR™ leadless pacemaker, including the first reported in person trans-baffle delivery of this device and implantation into a single ventricle heart. Our cases are a 30-year-old male with single ventricle physiology and Fontan circulation, found incidentally to have complete heart block (CHB) and a 48-year-old female with a Fontan circulation who had CHB and a history of syncope. These cases demonstrate a variety of venous access routes and add to the existing data of leadless pacemaker implantation in single ventricle physiology. The very long battery life of these devices make them ideal for patients with difficult venous access with bradycardia pacing requirement with low pacing burden.

**Discussion:**

Leadless pacemakers offer a valuable alternative to transvenous pacing leads for providing bradycardia pacing support in this patient population.

**Learning points**

- The adult congenital heart disease population can have unique and complex anatomy. A patient-centred approach to pacing strategies should be employed to get the early decisions right.
- Leadless pacemakers offer a safe pacing option with some devices providing very long battery life, minimising future procedures and saving venous access for future transvenous options.

## Introduction

We present two cases of novel use of the Abbott AVEIR™ leadless pacemaker, including the first reported trans-baffle delivery of this device and implantation into single ventricle physiology patients.

Our first case is of a 30-year-old male with double outlet right ventricle who had undergone a modified atrio-pulmonary Fontan procedure with intraatrial tunnel and was found to have an incidental finding of complete heart block (CHB). Our second case is of a 48-year-old female with hypoplastic right ventricle with left atrial isomerism who had previously undergone Kawashima procedure, who had CHB and a history of syncope. Both patients required complex baffle punctures to deliver the device, with Patient 1 requiring a puncture through atrial baffle tissue and Patient 2 requiring superior venous access with the device deployed through a puncture via the superior cavo-pulmonary shunt.

Given the low pacing burden, these patients have an estimated 25 and 23.8 years of battery life, respectively, on their device. These cases add to the knowledge base of the use of leadless pacemakers and demonstrate novel approaches for the adult congenital heart disease population who may have challenging anatomy.

## Summary figure

**Figure:**
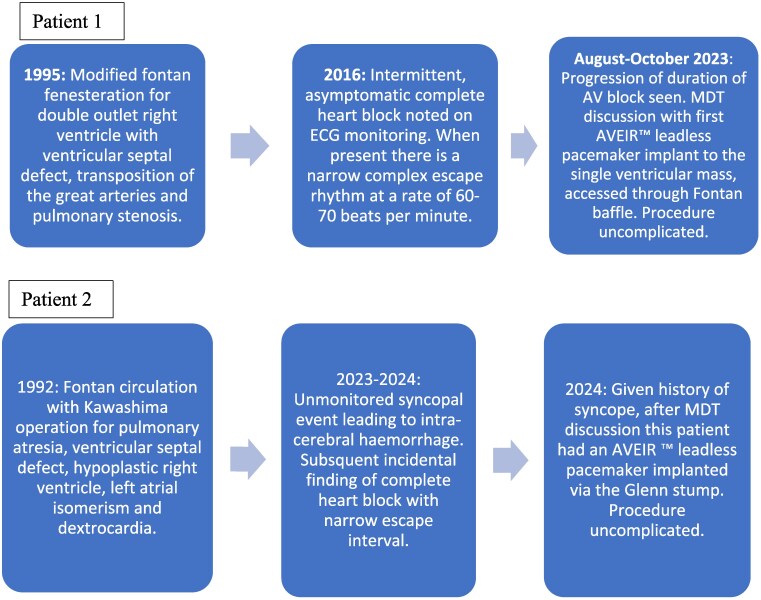


### Patient 1

**Figure 1:**
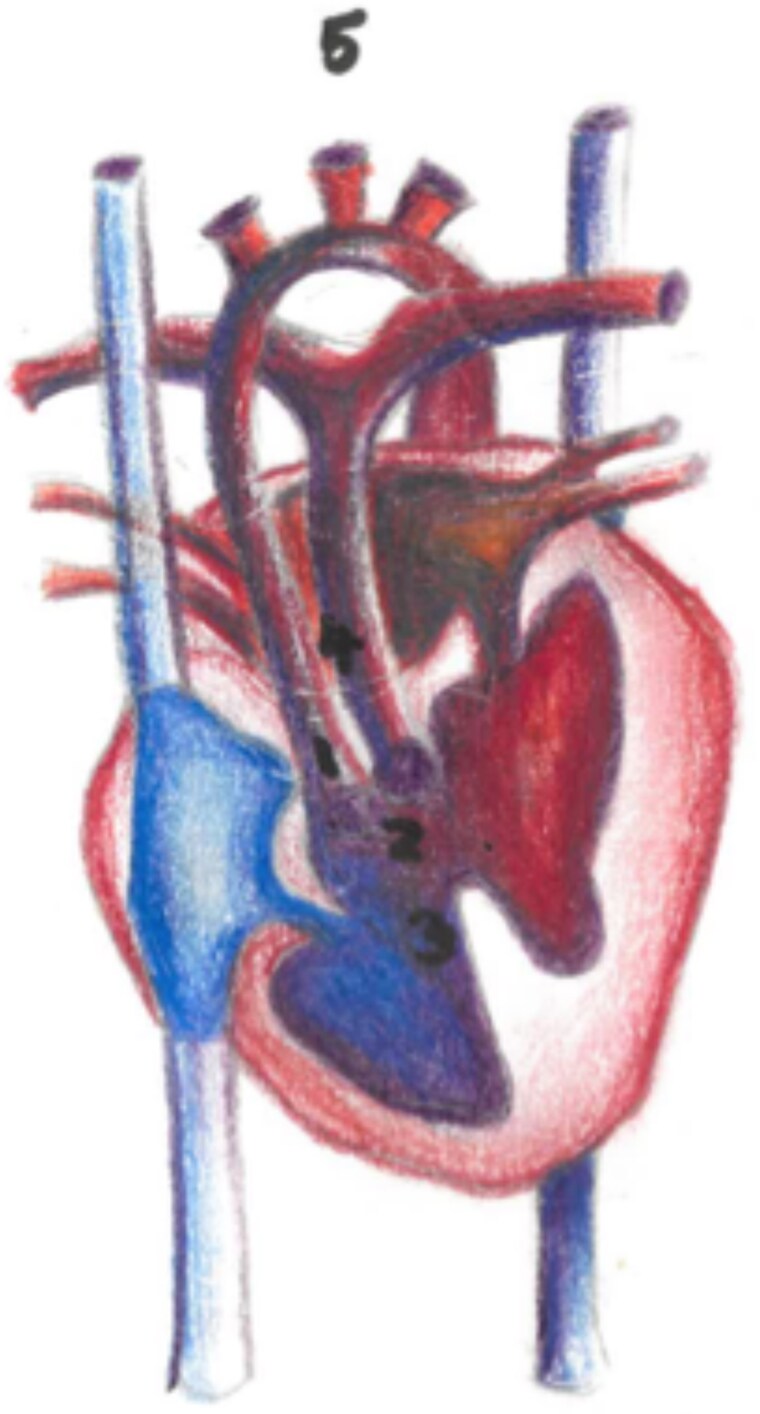
Patient 1 unoperated anatomy. (1) Pulmonary stenosis, (2) double outlet right ventricle, (3) VSD, (4) TGA, (5) bilateral SVC.

A 30-year-old male with complex congenital heart disease involving a double outlet right ventricle, transposition of the great arteries, ventricular septal defect (VSD), pulmonary stenosis, and bilateral superior vena cava (SVC) (*Figure 1*). Previous surgical intervention had taken place at age 2 consisting of a modified Fontan fenestration with anastomosis of the left SVC to left pulmonary artery (PA), right atrial appendage to right PA and the creation of an intraatrial tunnel.

During routine follow-up, an incidental finding of intermittent asymptomatic CHB was made and kept under close monitoring for several years. A 12-lead electrocardiogram recorded a narrow QRS of 106 ms and a ventricular escape rate of 64 b.p.m. (beats per minute). Ambulatory rhythm monitoring in August 2023 showed CHB throughout a 24-h recording; with a variable heart rate of between 30 and 64 bpm, the average heart rate was 42 bpm. The progression in duration of CHB over time and a reduction in ventricular rate prompted discussion regarding pacemaker implantation. It was noted that this patient was reported no exercise incapacity and no symptoms of presyncope or syncope.

After multi-disciplinary team (MDT) discussion, it was felt pacing should be offered given the now permanent CHB, reducing average ventricular rate and single ventricle physiology. Transvenous, surgical epicardial, and leadless pacing options were discussed, with the leadless option being the favored approach by the patient, a decision that was supported by the MDT to allow preservation of transvenous options for the future.

**Figure 2:**
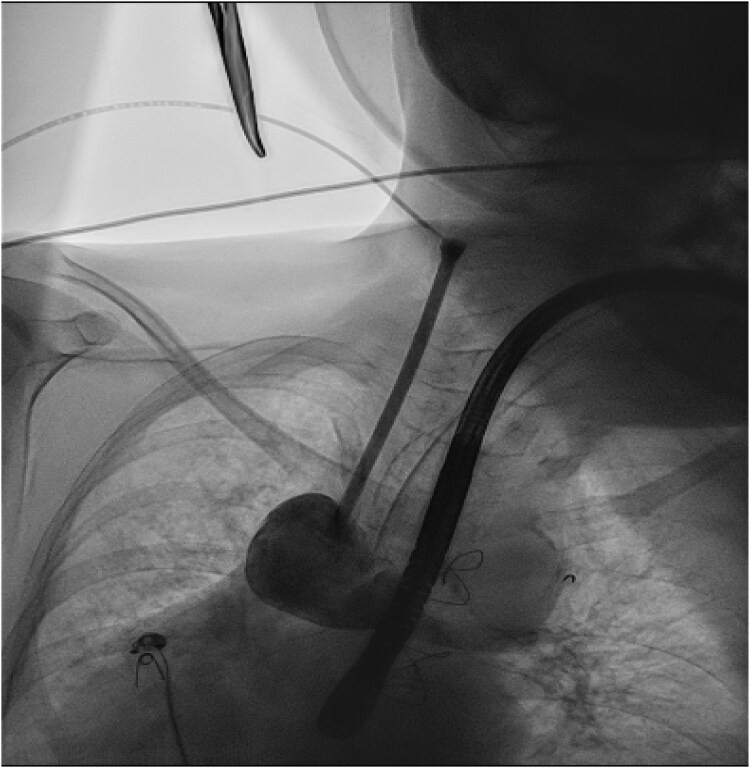
3D TOE echocardiogram image of RF needle puncturing Fontan baffle.

**Figure 3:**
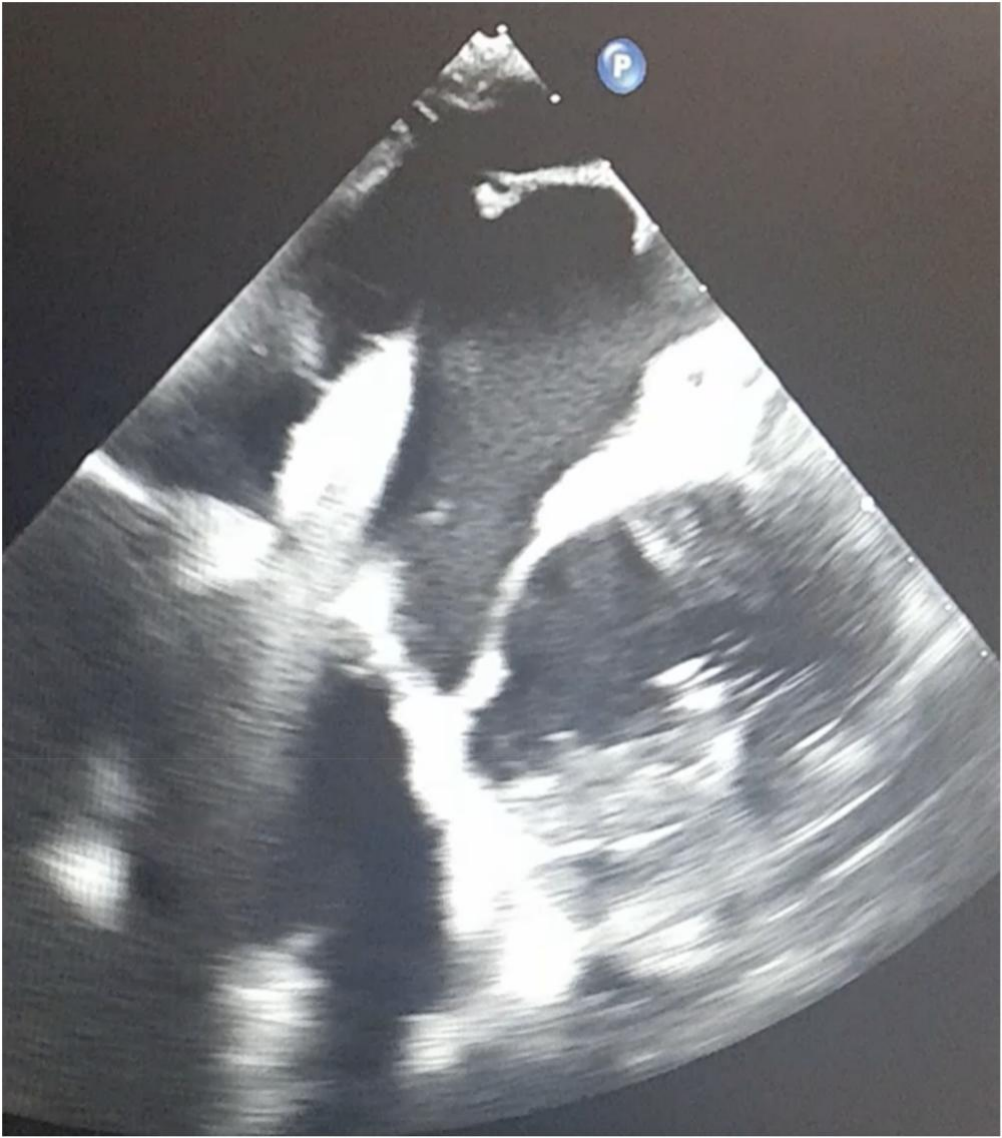
TOE imaging showing a 27Fr sheath passing through the baffle and into the pulmonary venous atrium.

**Figure 4:**
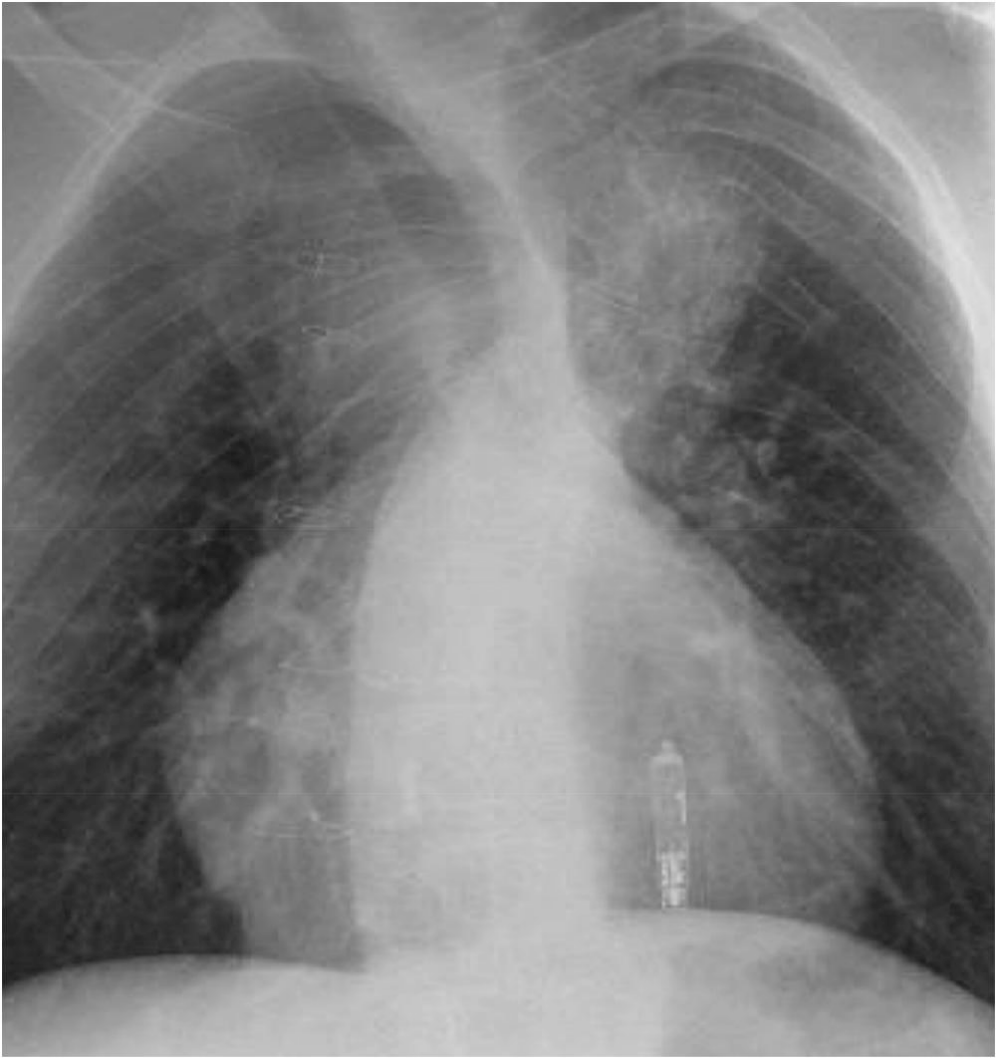
Post procedural chest x-ray confirming position of AVEIR™ device in the cardiac silhouette.

The procedure was carried out under general anesthetic with transoesophageal (TOE) echocardiographic support. Using ultrasound, a 9Fr sheath was placed in the right femoral vein. Angiography confirmed suitable iliac size, and then short sheath was upgraded to an Abbott Swartz™ LAMP™ 45 transeptal guiding introducer. Using a bayliss C-0 radiofrequency (RF) needle, under TOE support, the posterior baffle tissue was punctured (*Figure 2*). After confirmation of needle crossing, an Amplatzer super stiff J-wire was taken into the left upper pulmonary vein and then an 8 mm Boston Scientific Mustang™ balloon dilatation catheter was taken over this and inflated to 10 atmospheres to create a fenestration. The 27Fr delivery sheath was then advanced across the baffle hole using the super stiff wire (*Figure 3*) and into the pulmonary venous atrium. Here the device catheter was advanced out of the delivery sheath then rotated anteriorly and curved to cross the AV valve and into the main ventricular mass. After advancing the catheter to the ventricular apex, radiopaque contrast showed confirmation of a suitable ventricular position and an Abbott AVEIR™ single chamber leadless device was deployed onto the ventricular wall and released without complication. Post procedural chest x-ray (*Figure 4*) and device interrogation were satisfactory. The patient was discharged home the following day and a subsequent pacing check at 6 weeks confirmed estimated battery longevity at >25 years at the current pacing burden with good sensing and a threshold of <0.25 v at 0.4 ms.

### Patient 2

**Figure 5:**
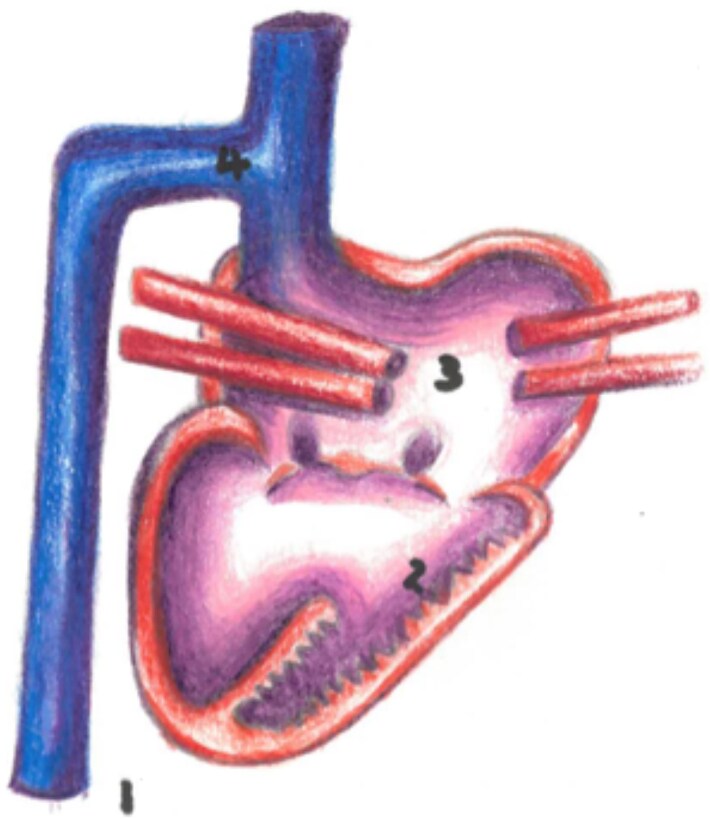
Patient 2 unoperated anatomy. (1) Dextrocardia, (2) Hypoplastic Right Ventricle, (3) Left Atrial Isomerism, (4) SVC with azygos continuation.

A 48-year-old female patient with pulmonary atresia, VSD, hypoplastic right ventricle, left atrial isomerism, and dextrocardia (*Figure 5*) had previously undergone a Kawashima operation at 17 years old. The patients’ condition was complicated by multiple intrapulmonary microvascular AV fistulas and veno-arterial collaterals leading to declining hypoxaemia and haemoptysis previously. A year prior to referral, she had had a cerebral haemorrhage following an unmonitored syncopal event.

At a routine outpatient follow-up at a neighboring hospital, she was found to have CHB and a narrow ventricular escape rhythm at 48 bpm. She was admitted for ECG monitoring, which continued to show CHB although the patient was largely asymptomatic. Given the severity of injury as a result of syncope and the presence of CHB, MDT discussion recommended a pacemaker implantation. It was agreed that a percutaneous first attempt was safer than redo-sternotomy due to the large volume of collaterals. Different pacing approaches were discussed, with a leadless pacemaker decided as the preferred option due to the lower infective risk and potentially lower embolic risk due to the parylene coating.

**Figure 6:**
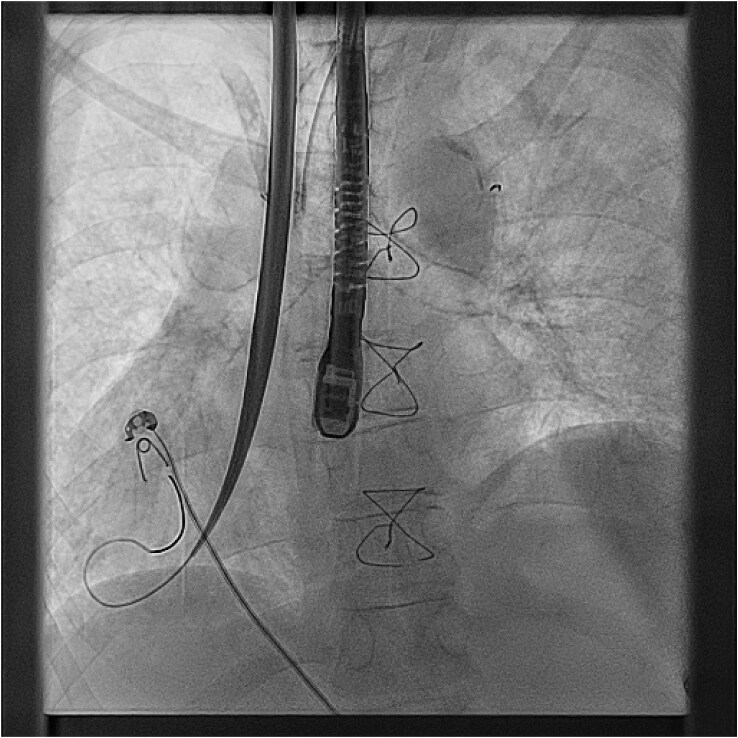
Right Internal Jugular access.

**Figure 7:**
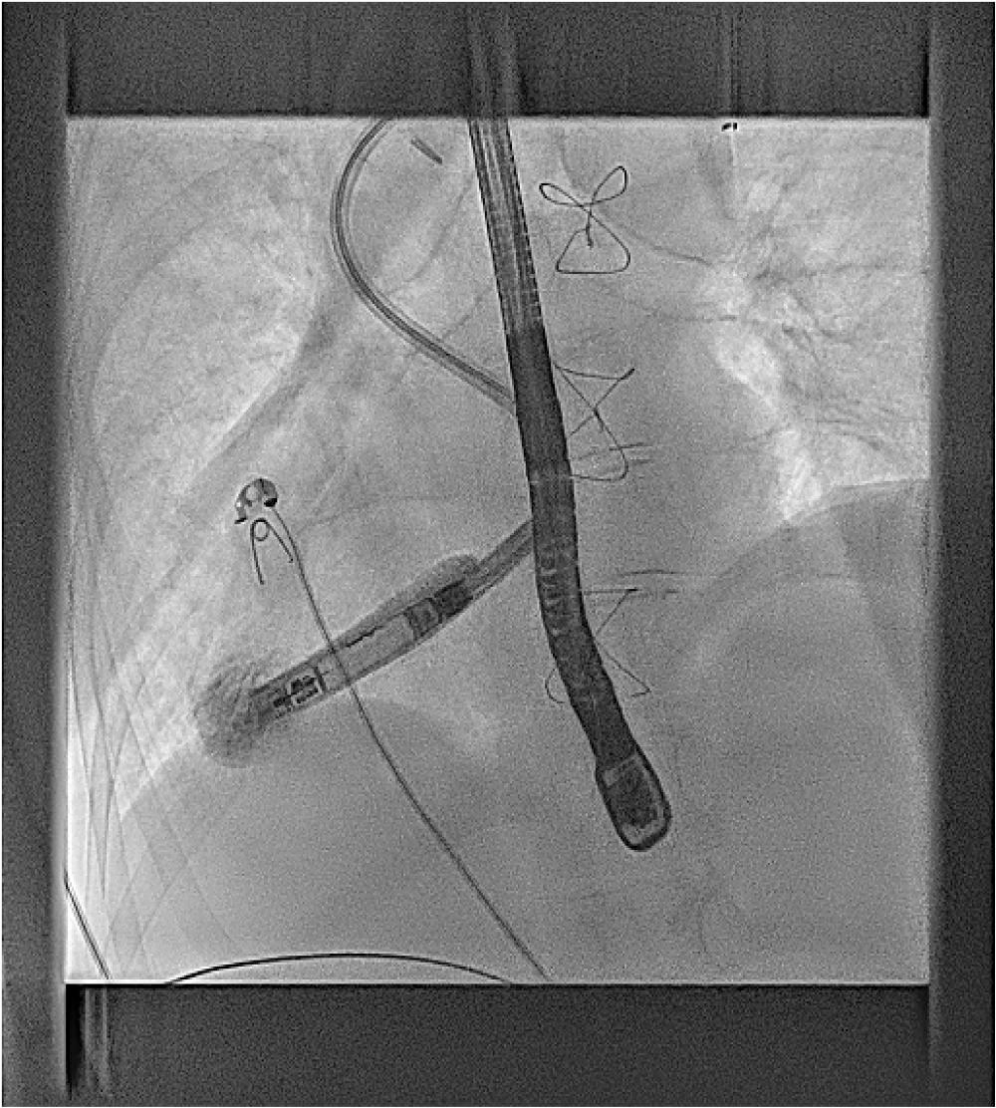
27Fr Aveir VVI Leadless pacemaker delivery system.

**Figure 8:**
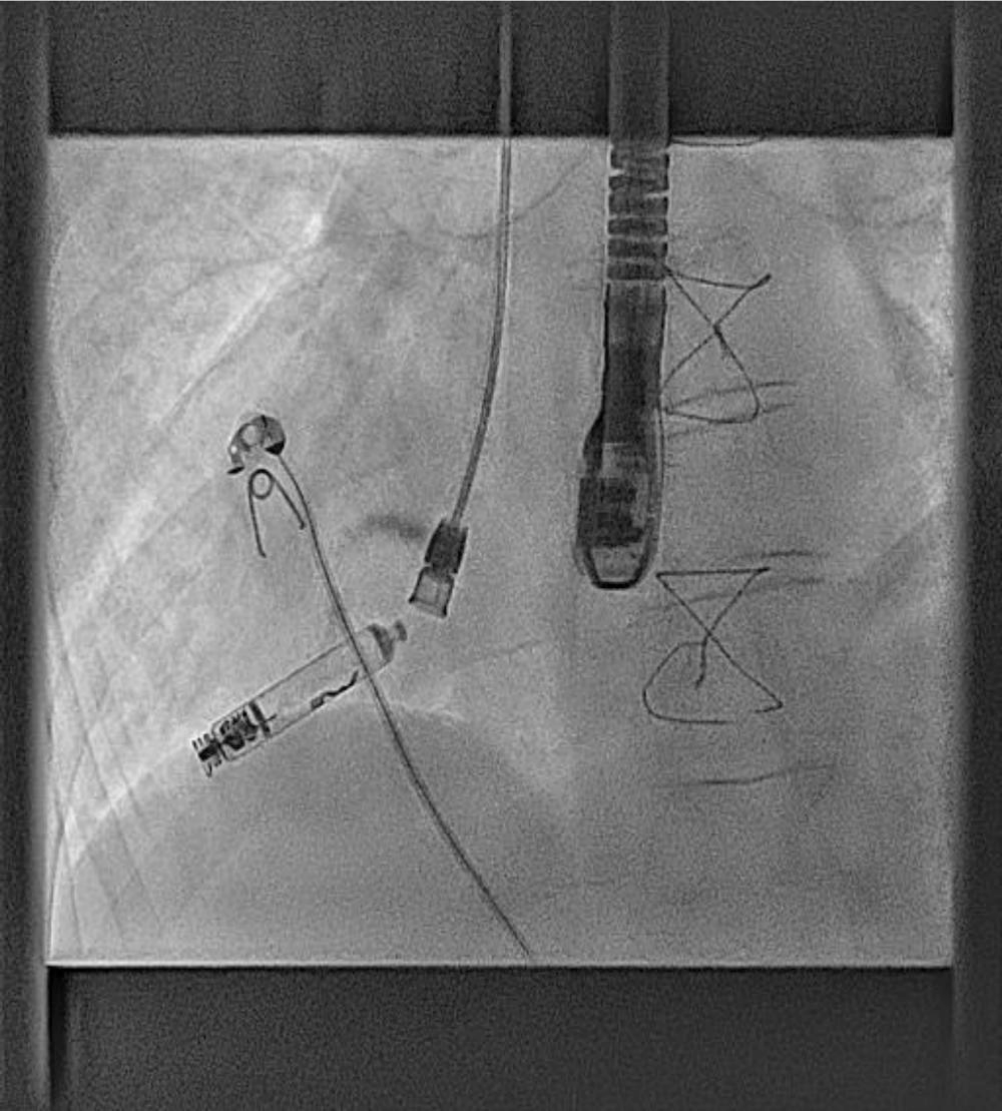
Positioning of device at ventricular apex.

Under general anaesthetic and with TOE imaging, an ultrasound-guided puncture to the right internal jugular vein (RIJ) (*Figure 6*) was made due to the lack of suitable femoral access due to left atrial isomerism. An SRO sheath and C0 RF needle were used to puncture the Glenn stump to access the atrial mass. A lunderquist wire was then advanced into the ventricular apex (*Figure 7*). The delivery tract was then upsized using a Coon’s dilator to allow for the AVEIR™ 27Fr delivery system to advance to the ventricular apex (*Figure 8*). The VVI AVEIR™ leadless pacemaker device was uncovered in the ventricular apex. The apex was chosen primarily, to ensure AV valve clearance.

The electrical pacing parameters were excellent in this position, with an R wave of 5 mv, impedance of 500 ohms, and a threshold of 0.5v@0.4 ms. The device was released without complication, and the RIJ access closed with pressure and a Z-stitch.

Post procedural chest x-ray and device interrogation were satisfactory. The patient was discharged home 2 days later, and a subsequent pacing check at 6 weeks confirmed estimated battery longevity at >23 years at the current pacing burden of 16% with good sensing and pacing parameters, comparable to those at implant.

## Discussion

The use of leadless pacemakers has become a well-established pacing alternative to the traditional transvenous pacemaker implant^1,2^ A key advantage being the lack of commitment to a transvenous lead and the associated complications that can develop over the lifetime of these devices implanted in the young population. Furthermore, leadless devices do not require SVC venous access, which can be absent or difficult to access in the adult congenital heart disease population.^3^ We also demonstrate that superior venous access is an alternative if anatomically required.

This AVEIR™ system is designed to provide VVI pacing support; however, there is a dual chamber system available to which an upgrade at a later stage can be considered if AV synchrony is required. These cases demonstrate how the leadless pacemaker can provide an excellent option in providing bradycardia backup support in patients who are likely to have a very small pacing burden initially, but in whom the safety of a device is desired.

Leadless devices such as the AVEIR™ have traditionally been utilized where there have been specific concerns, contraindications or barriers to a transvenous system including:

1. Occluded upper limb venous systems.
2. Concerns regarding infective risk.
3. Bioprosthethic tricuspid valve.
4. Desire to preserve upper limb venous access such as for dialysis.
5. Desire to preserve venous access in young patients with low pacing requirement.

In particular, in congenital heart disease, infective risk with pacemaker devices can be high and has been reported at varying levels up to 7.8%.^4^ In addition to infective risks, access difficulties such as with our cases make pacing in congenital heart disease potentially more technically challenging for operators.

The AVEIR™ was chosen in particular here due to the ability to deliver to a systemic ventricle with a screw in system for security, the extremely low infective rates seen with leadless devices^5^ and parylene coating, which is of low thrombogenicity.^6^

The major contraindications to delivery of a leadless system would be where a patient requires resynchronization or conduction system pacing for heart failure, active thrombosis in the access vessel, and need to cross a mechanical valve to deliver. Currently, leadless devices such as the AVEIR™ are not licensed for use in the systemic ventricle and are used here compassionately in challenging circumstances.

Long-term data for the AVEIR™ is still small, but there is data on retrieval of devices such as the AVEIR™ up to 9 years after implant^3^ and data from leadless devices in general out to and over 5 years is very promising.^7^

These cases also demonstrate a novel use of the device in the adult congenital population and the first reported in-person deployment in a single ventricle, systemic ventricle, and across a baffle. Whilst leadless devices have been used in a single ventricle population^8,9^ this is a first case series example of the AVEIR™ retrievable leadless device being used in this cohort of patients.

The AVEIR™ has potential advantages over other leadless devices as it is designed to be able to be retrieved at a later stage if required,^3,9^ has a long battery life, and has an active fix deployment mechanism.

The development of an atrial AVEIR™ leadless device could address the need for future AV synchrony, as this modular device can be added to allow communication and therefore AV sequential pacing and was of attraction in our case as the pacing requirement increases with time.

Patients with a Fontan circulation have higher requirements for pacing compared with their peers, either due to significant SA node dysfunction or AV block^10^ which may occur operatively, or because of congenital abnormalities such as L-transposition of the great arteries. Although pacing in young people is not ideal, it is often necessary in this cohort of patients, and devices that can provide optimal device longevity with smaller, less complex operative techniques are of interest, especially in light of the traditional reliance on epicardial pacing.

Leadless pacemakers have a device longevity of ∼5–15 years, similar to that of TV devices. The AVEIR™ system can have an estimated battery life of up to 25 years (demonstrated in this case series at first follow-up), when programmed to a minimal pacing setting (VVI@30 bpm) and with a minimal pacing burden. For these patients, a battery life of 25 years would mean perhaps one or even no further pacing procedures in their lifetime, further reducing the risk of future complications.

Despite having a larger battery capacity than other leadless pacemakers, one current limitation comes in the form of limited remote follow-up capabilities. The AVEIR utilizes a novel telemetry technology for two purposes, as it significantly reduces battery drain but also it also utilized for the i2i communication between the atrial and ventricular leadless devices. The technology used is conductive patient telemetry, meaning electrodes must be applied to the patient's chest to allow interrogation or programming of the device.

This means currently there is no remote follow-up of these patients, and all follow-up must be in person, though this may change in the future as new technology is released.^11^ Other leadless pacemakers use inductive telemetry, allowing for remote follow-ups.

In summary, the Abbott AVEIR™ remains an excellent option in providing pacing support in the complex congenital heart disease population.

## Patient’s perspective

Patient 1 was very afraid of having any pacing procedure, especially open surgery or having a pacing box under the skin, after this device was implanted, he reports to feeling ‘great’.

## Lead author biography


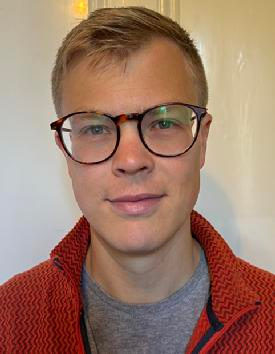


Senior Cardiac Devices Fellow at Liverpool Heart and Chest Hospital.

## Supplementary Material

ytaf146_Supplementary_Data

## Data Availability

The data underlying this article will be shared on reasonable request to the corresponding author.
