# Supplementary material for: Case report demonstrating novel approaches for leadless pacemaker implantation in the single ventricle heart
Source: Eur Heart J Case Rep. 2025 Mar 28;9(4):ytaf146. doi: 10.1093/ehjcr/ytaf146 (PMC12038896; doi:10.1093/ehjcr/ytaf146)
Supplement: ytaf146_Supplementary_Data [file ytaf146_supplementary_data.docx]

Suppplement 1: Fontan with atrial baffle. (1) Intra-atrial baffle, (2) Anastomosis of SVC to RPA.
